# Supplementary material for: Drug repurposing for aging research using model organisms
Source: Aging Cell. 2017 Jun 16;16(5):1006–15. doi: 10.1111/acel.12626 (PMC5595691; doi:10.1111/acel.12626)
Supplement: Supplementary file 7 — Data S1 Zip‐Archive of all report cards. [file ACEL-16-1006-s007.zip › RC_MAH.pdf]

MAH

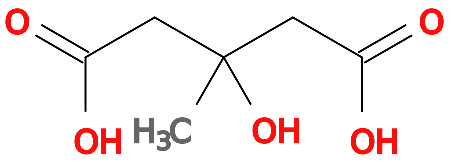

Database identifiers

ChEMBLCompound	CHEMBL50444
DrugBank	DB04377
CHEBI	16831
eMolecules	520938

Ranking

	Rank	Score
Drosophila	378/697	0.392
C. elegans	243/591	0.224

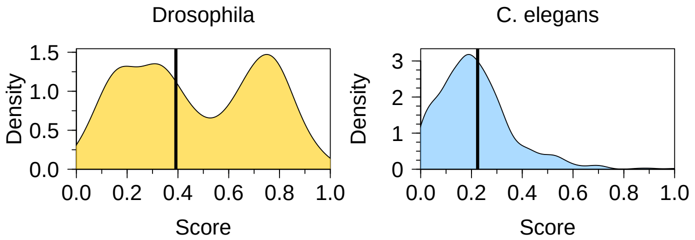

	Ageing implication	Domain conservation	Binding site conservation	Binding affinity	Bioavailability	Lipinski	Promiscuity	Purchasability	Drug approval	**Total**
Drosophila	0.792	0.912	1.0	0.334	(0.9)	0.0	-0.0	0.1	0.075	0.392
C. elegans	0.792	0.831	1.0	0.334	0.226	0.0	-0.0	0.1	0.075	0.224

Names

- **3-hydroxy-3-methylglutaric acid**
- 3-Hydroxy-3-methylpentanedioic acid
- beta-hydroxy-beta-methylglutaric acid
- Dicrotalic acid
- Meglutol

Roles

plant metabolite, human metabolite, antimetabolite, anticholesteremic drug, EC 1.1.1.34/EC 1.1.1.88 (hydroxymethylglutaryl-CoA reductase) inhibitor

Status

Approved drug (according to ChEMBL)	No
Classification (according to DrugBank)	experimental
Number of Rule of 5 violations	0
Binding affinity to original target in log units (RF-Score prediction)	4.31
Burns *C. elegans* bioavailability prediction	-3.65

Compound Target Characteristics

3-hydroxy-3-methylglutaryl-coenzyme A reductase

Best gene implication in ageing for this target family came from gene P51639 annotated in UniProt release 2014_02. Annotation GO 7568 (aging) was Inferred from Expression Pattern

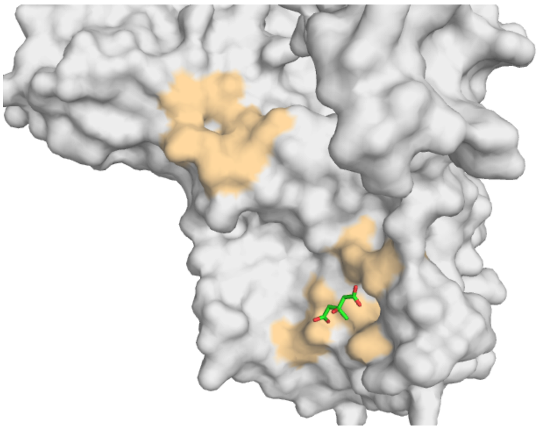

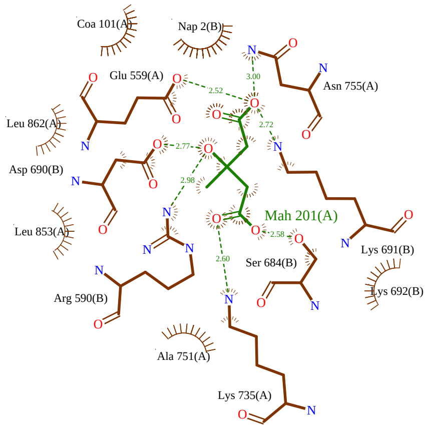

protein	amino acids contacts (binding site)
PDB:1dq8:chainA:P04035	E R M S N D K K K A N L L
PDB:1dqa:chainA:P04035	E R M S N D K K K A N L L
sp:P04035:HMDH_HUMAN	E R M S N D K K K A N L L
sp:P51639:HMDH_RAT	E R M S N D K K K A N L L
sp:Q01237:HMDH_MOUSE	E R M S N D K K K A N L L
tr:E1JIU8:E1JIU8_DROME	E R M S N D K K K A N L L
tr:A9UN11:A9UN11_DROME	E R M S N D K K K A N L L
sp:P14773:HMDH_DROME	E R M S N D K K K A N L L
tr:Q19207:Q19207_CAEEL	E R M S N D K K K A N L L
sp:P12684:HMDH2_YEAST	E R M S N D K K K A N L L
sp:P12683:HMDH1_YEAST	E R M S N D K K K A N L L

	whole protein		domain-based		contact-based	
protein	ident	simil	ident	simil	ident	simil
PDB:1dq8:chainA:P04035	1.0	1.0	1.0	1.0	1.0	1.0
PDB:1dqa:chainA:P04035	1.0	1.0	1.0	1.0	1.0	1.0
sp:P04035:HMDH_HUMAN	1.0	1.0	1.0	1.0	1.0	1.0
sp:P51639:HMDH_RAT	0.93	0.98	0.93	0.98	1.0	1.0
sp:Q01237:HMDH_MOUSE	0.93	0.98	0.95	0.99	1.0	1.0
tr:E1JIU8:E1JIU8_DROME	0.44	0.74	0.59	0.83	1.0	1.0
tr:A9UN11:A9UN11_DROME	0.44	0.74	0.58	0.83	1.0	1.0
sp:P14773:HMDH_DROME	0.44	0.74	0.59	0.83	1.0	1.0
tr:Q19207:Q19207_CAEEL	0.24	0.43	0.49	0.76	1.0	1.0
sp:P12684:HMDH2_YEAST	0.3	0.63	0.6	0.84	1.0	1.0
sp:P12683:HMDH1_YEAST	0.29	0.61	0.59	0.81	1.0	1.0

Hmgcr (FBgn0263782) associated phenotypes

cold sensitive, developmental rate defective, germline clone, heat sensitive, lethal - all die before end of second instar larval stage, partially, partially lethal - majority die, rescuable maternal effect, segment polarity phenotype, small body, somatic clone, some die during pupal stage, some die during second instar larval stage, some die during third instar larval stage

(Information from FlyBase)

Hmgcr (UniProt:E1JIU8) annotation

Pathway: Metabolic intermediate biosynthesis; (R)-mevalonate biosynthesis; (R)-mevalonate from acetyl-CoA: step 3/3. (RuleBase:RU361219).

Subcellular location: Endoplasmic reticulum membrane (RuleBase:RU361219); Multi-pass membrane protein (RuleBase:RU361219).

(Information from UniProt)

Hmgcr (UniProt:A9UN11) annotation
Pathway: Metabolic intermediate biosynthesis; (R)-mevalonate biosynthesis; (R)-mevalonate from acetyl-CoA: step 3/3. (RuleBase:RU361219).
Subcellular location: Endoplasmic reticulum membrane (RuleBase:RU361219); Multi-pass membrane protein (RuleBase:RU361219).

(Information from UniProt)

Hmgcr (UniProt:P14773) annotation
Function: Synthesis of mevalonate for the production of non-sterol isoprenoids, which are essential for growth differentiation. Provides spatial information during embryogenesis to guide migrating primordial germ cells (the pole cells) from the ectoderm to the mesoderm. Also required for association of the pole cells with the gonadal mesoderm. (PubMed:9853754).
Enzyme regulation: The activity of HMG-CoA-reductase is suppressed by exogenous mevalonate. (PubMed:3136321).
Pathway: Metabolic intermediate biosynthesis; (R)-mevalonate biosynthesis; (R)-mevalonate from acetyl-CoA: step 3/3.
Subcellular location: Endoplasmic reticulum membrane; Multi-pass membrane protein.
Tissue specificity: Highly expressed in embryonic gonadal mesoderm, where expression is initially broad, and then becomes restricted to a segmental pattern at stage 11. Expression is then further restricted to a cluster of cells in each of parasegments 10, 11 and 12, corresponding to the developing gonadal mesoderm. Not expressed in pole cells. (PubMed:9853754).

(Information from UniProt)

hmgr-1 (WBGene00017268) associated phenotypes
lethal, sterile

(Information from WormBase)

hmgr-1 (UniProt:Q19207) annotation
Pathway: Metabolic intermediate biosynthesis; (R)-mevalonate biosynthesis; (R)-mevalonate from acetyl-CoA: step 3/3. (RuleBase:RU361219).
Subcellular location: Endoplasmic reticulum membrane (RuleBase:RU361219); Multi-pass membrane protein (RuleBase:RU361219).

(Information from UniProt)
